# Acute Effects of Sex Steroid Hormones on Susceptibility to Cardiac Arrhythmias: A Simulation Study

**DOI:** 10.1371/journal.pcbi.1000658

**Published:** 2010-01-29

**Authors:** Pei-Chi Yang, Junko Kurokawa, Tetsushi Furukawa, Colleen E. Clancy

**Affiliations:** 1 Department of Pharmacology, University of California, Davis, Davis, California, United States of America; 2 Department of Bio-informational Pharmacology, Medical Research Institute, Tokyo Medical and Dental University, Tokyo, Japan; University of California, San Diego, United States of America

## Abstract

Acute effects of sex steroid hormones likely contribute to the observation that post-pubescent males have shorter QT intervals than females. However, the specific role for hormones in modulating cardiac electrophysiological parameters and arrhythmia vulnerability is unclear. Here we use a computational modeling approach to incorporate experimentally measured effects of physiological concentrations of testosterone, estrogen and progesterone on cardiac ion channel targets. We then study the hormone effects on ventricular cell and tissue dynamics comprised of Faber-Rudy computational models. The “female” model predicts changes in action potential duration (APD) at different stages of the menstrual cycle that are consistent with clinically observed QT interval fluctuations. The “male” model predicts shortening of APD and QT interval at physiological testosterone concentrations. The model suggests increased susceptibility to drug-induced arrhythmia when estradiol levels are high, while testosterone and progesterone are apparently protective. Simulations predict the effects of sex steroid hormones on clinically observed QT intervals and reveal mechanisms of estrogen-mediated susceptibility to prolongation of QT interval. The simulations also indicate that acute effects of estrogen are not alone sufficient to cause arrhythmia triggers and explain the increased risk of females to Torsades de Pointes. Our results suggest that acute effects of sex steroid hormones on cardiac ion channels are sufficient to account for some aspects of gender specific susceptibility to long-QT linked arrhythmias.

## Author Summary

It is well known that female gender is an independent risk factor for some types of cardiac arrhythmias. However, it has been difficult to determine how much of a role physiological concentrations of circulating sex steroid hormones play in gender linked arrhythmia susceptibility because the cardiac system is so extraordinarily complex. Here we employ a computational strategy, based on experimental measurements, to tease out the individual contributions of estrogen, progesterone and testosterone on cardiac electrical behavior and then make predictions about their effects in combination and in the presence of drugs. The computational models convincingly reproduce observed fluctuations of QT intervals (as recorded on the ECG (electrocardiogram), the QT interval reflects the time period between ventricular excitation and relaxation) through the menstrual cycle in females and effects of testosterone on ECG parameters. Our simulations also predict that testosterone and progesterone are protective against drug-induced arrhythmias, while estrogen likely exacerbates the breakdown of normal cardiac electrical activity in the presence of QT-prolonging drugs.

## Introduction

In the past decade, studies have suggested that female gender is an independent risk factor for long-QT (LQT) dependent cardiac arrhythmias [1]–[3]. Since the differences in QT intervals in males and females appear from the time of puberty [4],[5], sex steroid hormone effects on cardiac repolarization have been implicated. Clinical studies have found no difference in QT interval in male and female children, but shorter QT intervals in men versus women under age 50 [4]. The international Long QT syndrome (LQTS) registry 1998 reported that females had higher risk of a first cardiac event between 15 and 40 years [6]. Moreover, clinical findings observed that more than 68% of drug-induced torsade de pointes (TdP) occur in women [7]–[9].

It is known that one way that sex steroid hormones cause functional physiological changes is via transcriptional regulation. Sex hormones may bind to sex hormone receptors and then translocate into the nucleus. In the nucleus, a ligand-bound sex hormone receptor acts a transcription factor by binding to the promoter region of genes containing a hormone responsive element (HRE), leading to regulation of gene expression. For example, in the heart, lipocalin-type prostaglandli D synthase (L-PDGS) has been found to be transcriptionally upregulated by estradiol and estrogen receptor (ER) [10]. This genomic action requires several hours before the effects can be observed. In addition to the genomic pathway, sex steroid hormones may induce a rapid activation of mitogen-activated protein kinase (MAPK) leading to transcription factor activation [11],[12] as well as activation of membrane bound endothelial nitric oxide synthase (eNOS) [13],[14].

Interestingly, recent studies have demonstrated that sex steroid hormones may also act acutely and rapidly modulate cardiac ion channel activity directly via a PI3K/Akt/eNOS pathway [15]–[17]. Testosterone induced phosphorylation of the Ser/Thr kinase Akt and eNOS leads to NO synthase 3 (NOS3) activation and production of nitric oxide (NO) [15]. NO leads to s-nitrosylation of cysteine residues on the channel underlying the slow delayed rectifier K^+^ current (I_Ks_) [17]. L-type Ca^2+^ current (I_Ca,L_) is conversely suppressed by NO via a cGMP dependent pathway. Regulation of I_Ks_ and I_Ca,L_ by testosterone is dose-dependent [15] and leads to shortening of action potential duration (APD) [15] and QT intervals [18]–[20]. In adult men, the serum testosterone level is reported to be 10 to 35 nM [21], however circulating levels of testosterone begin to decline in men as young as 40 [22]. QT intervals are shorter in adult men than in adult women until around the age of 50 [4], suggesting a likely role for circulating testosterone.

In females, progesterone fluctuates through the menstrual cycle. The reported serum progesterone level is 2.5 nM in the follicular phase and 40.6 nM in the luteal phase [23]. It was recently shown by Nakamura et al. that progesterone increases I_Ks_ current through the NO production pathways and prevents cAMP-enhancement of I_Ca,L_ [16].

The apparent result of acute effects of progesterone and testosterone is to shorten ventricular repolarization and diminish incidence of arrhythmias [15],[16],[20],[24]. Recently, experiments have suggested protective effects of testosterone against arrhythmia. *In vivo* experiments show that orchiectomized male rabbits treated with dihydrotestosterone (DHT) had shorter QT interval and APD_90_ compared to non-DHT treated rabbits [18],[20]. Also, experiments in testosterone treated female animals have shown that DHT reduces drug-induced arrhythmia by dofetilide [24].

The acute effects of estradiol result in suppression of human ether-a-go-go-related gene (hERG) underlying the rapid delayed rectifier current (I_Kr_) by directly binding to the channel, altering channel kinetics and reducing current [25]. Kurokawa and co-workers showed that 17β-estradiol (E2) increases the channel rate of closure (deactivation) and lessens repolarizing current. They also showed that in the presence of E2, hERG is more sensitive to block by drugs. The group proposed that aromatic centroid of E2 may be responsible for increasing the sensitivity of hERG block by E4031 via interaction with the aromatic side chain of Phe^656^ and aromatic rings of the hERG blocker. Because 1) the concentration of E2 is not constant through the menstrual cycle, but rather fluctuates from the peak follicular phase serum E2 level of 1 nM to 0.7 nM in the luteal phase, and 2) E2 has dramatic effects on sensitivity to hERG block within this range, it stands to reason that susceptibility to drug-induced arrhythmia by hERG block may vary through the menstrual cycle.

Although studies have shown that female hormones estradiol and progesterone have opposite effects on cardiac repolarization: E2 prolongs QT intervals, and progesterone reduces QT interval [16],[25],[26], the question of whether normal hormonal fluctuations are sufficient to account for variability in QT during the menstrual cycle in not known. Neither are the effects of physiological concentrations of hormones on arrhythmia susceptibility well understood. Some studies do report that dynamic fluctuations in QT intervals during the menstrual cycle are related to changes in susceptibility to TdP risk [27],[28]. Other studies in postmenopausal women also suggest the importance of female hormones as estrogen hormone replacement therapy prolongs QT intervals and increases arrhythmia risk [26],[29],[30]. Other data have not found marked fluctuation in QT interval during specific phases of the menstrual cycle [28],[31],[32]. Burke et al., (1997) found that the corrected QT (QT_c_) interval does not significantly change through menstrual cycle in pre-menopausal women; however, QT_c_ is reduced in the luteal phase after autonomic blockade [31]. A study of drug-induced QT prolongation during the menstrual cycle observed that QT_c_ did not vary during the menstrual cycle, but QT_c_ shortening was more pronounced in the luteal phase with ibutilide application [28]. Nonetheless, both the clinical and experimental data suggest that women have both longer QT intervals than men and are more likely to develop long-QT dependent arrhythmias and TdP arrhythmias [9],[28]. Women are especially susceptible to increased arrhythmia risk in response to QT-prolongation drugs [9],[28],[33],[34].

It is a major challenge to specifically determine the relationship between sex steroid hormones and arrhythmia susceptibility in males and females since the cardiac system is extraordinarily complex. In order to attribute risk to a particular parameter, in this case physiologically relevant concentrations of sex steroid hormones, the specific effect must be studied in isolation without other perturbations to the system. This is the strength of the computational approach that we employ. In the present study, we focus on acute effects of sex steroid hormones on cardiac ion channel targets. We use guinea pig models that incorporate the effects of hormones measured experimentally from guinea pig, and then can test these changes specifically within the complex cellular and tissue milieu.

Importantly, we use the model to make predictions about the effects of physiological concentrations of sex steroid hormones on gender specific cardiac physiology parameters and arrhythmia susceptibility. Some recent experimental studies investigating functional effects of sex hormones on cardiac function have utilized hormone concentrations in the micromolar range that is orders of magnitude higher than the nanomolar physiological circulating concentration of E2 [35]. This is a critical consideration because micromolar concentrations of E2 are apparently cardioprotective via effects on L-type Ca^2+^ current (I_Ca,L_). Although high hormone concentrations may be relevant during phases such as pregnancy, a recent study showed that E2 at 1 nM did not have significant effects on I_Ks_ or I_Ca,L_ [25]. Our model simulations reproduce observed fluctuations of QT through the menstrual cycle in females in both cell and tissue-level. Simulations also predict that effects of testosterone and progesterone on ion channels hasten repolarization and protect from drug-induced arrhythmias.

## Results

### Sex steroid hormones effects on cardiac ion channels

To investigate the acute effects of sex steroid hormone on cardiac electrophysiology and arrhythmia susceptibility, we developed a computational model that mimicked the conditions employed experimentally so that we could directly validate our model by comparison to experimental measurements. Experiments were conducted in isolated ventricular myocytes from Langendorff-perfused adult female guinea pigs, so that they were free of endogenous neuronal and hormonal effects. The isolated cells were then incubated with human physiological concentrations of hormones for 10 min. and the effects of hormones on cardiac ion channels were measured. A range of cardiac ion channels were screened for functional changes induced by sex steroid hormones, but acute effects of progesterone were found only to modify I_Ks_ [16] while testosterone primarily increased I_Ks_ and inhibited I_Ca,L_ [15]; acute E2 treatment only significantly suppressed I_Kr_ current [25]. We utilized the experimentally observed effects of physiological concentrations of sex-steroid hormones in adult women and men and incorporated these functional changes into our computational models (described in detail in Supplemental Text S1).

**Figure 1:**
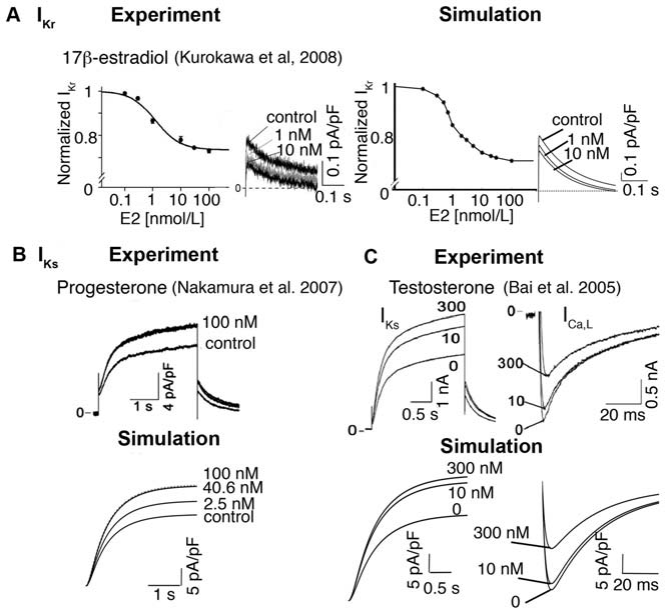
Effects of sex-steroid hormones E2, progesterone, and testosterone on cardiac ion channels. (A) Dose-dependence curves are shown for experimental (left traces) and simulated (right traces) inhibition of I_Kr_ current by E2. The simulated I_Kr_ tail currents (right) compared to experimentally measured I_Kr_ (left) at −40 mV following depolarization to a test potential = +20 mV in the absence (control) or presence of E2 (1 and 10 nM). (B) I_Ks_ was experimentally recorded at a test potential of +50 mV from a holding potential of −40 mV with 0 nM and 100 nM progesterone (top traces). Simulated (lower races) I_Ks_ are shown in the presence of 0 nM (control case), 2.5 nM (follicular phase), 40.6 nM (luteal phase) and 100 nM progesterone during a voltage pulse from −40 mV to +50 mV. (C) I_Ks_ (left panels) were elicited by 3.5-s test pulses to +50 mV from a holding potential of −40 mV (experiment — top traces and simulation — lower traces) in the absence and presence of testosterone (10 nM and 300 nM). The effect on I_Ca,L_ (right panels) from experimental data (top traces) and simulated results (lower traces) during a voltage step from −40 mV to 0 mV under control condition (0 nM), 10 nM and 300 nM testosterone.

Experiments [25] show that E2 primarily affects the conductance of I_Kr_, and has a minor, but measurable and significant effect on slowing channel activation kinetics. To simulate the experimentally observed I_Kr_ current reduction by E2 (Figure 1A – right), we scaled the I_Kr_ conductance and incorporated the minor effects of E2 on the voltage dependence (not shown) of I_Kr_ in the model (Figure 1A – left). E2 at 1 nM reduced I_Kr_ tail current in a dose-dependent manner, but did not affect the time course of tail current decay (Figure 1A).

Unlike the direct effects of E2 on I_Kr_, progesterone modulates the I_Ks_ through non-genomic activation of eNOS. We used experimental data [16] (Figure 1B – left traces) to scale the conductance of ionic currents in the model to incorporate effects of progesterone on I_Ks_. Progesterone-induced I_Ks_ enhancement is concentration-dependent as shown in Figure 1B. Experimentally recorded and simulated dose-response curves for progesterone effects on I_Ks_ tail current amplitude is shown in Supplemental Figure S1. I_Ks_ current was simulated with different concentrations corresponding to progesterone concentrations at various points in the menstrual cycle (0 nM – control case, 2.5 nM – follicular phase, 40.6 nM – luteal phase and 100 nM - maximal experimental concentration) during a voltage pulse from −40 mV to +50 mV. Note that the effect of progesterone on I_Ks_ is nearly saturated at a concentration of 40.6 nM, corresponding to the peak value during the luteal phase of the menstrual cycle (indicated by the near overlay of the 100 nM curve).

Like progesterone, testosterone modifies cardiac ion channels comprising I_Ks_ and I_Ca,L_ via eNOS production of NO. We used the same method as above to incorporate experimental ratios of control conductance for testosterone. Dose-dependent effects of testosterone on I_Ks_ enhancement and I_Ca,L_ suppression are shown in Figure 1C for experiments (top) and simulated currents (lower panels). Simulated I_Ks_ and I_Ca,L_ are compared to experimentally recorded guinea pig I_Ks_ and I_Ca,L_ using the same protocol. Cells were depolarized to test potential +50 mV for 3.5 seconds and then repolarized to −40 mV to record I_Ks_. I_Ca,L_ was experimentally recorded during a voltage step from −40 mV to 0 mV. Testosterone strongly enhances I_Ks_ current (Figure 1C – left traces) at 10 nM while high concentrations of testosterone (300 nM) markedly suppress I_Ca,L_ (Figure 1C – right traces).

### Simulated effects of hormones on action potentials in single cells

**Figure 2:**
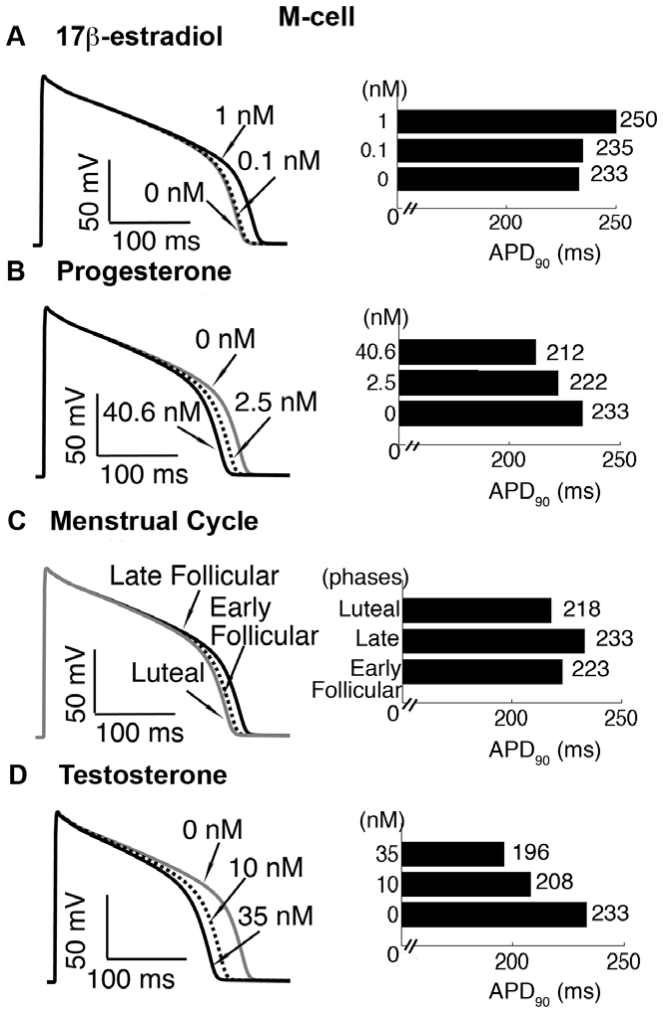
Simulated effects of sex hormones on cardiac action potentials. The APD for each concentration of sex-steroid hormone is indicated for the 50^th^ paced beat at a cycle length of 1000 ms in single M-cells. (A)–(B) Simulated APD in the presence of E2 (0.1 and 1 nM) and progesterone (2.5 and 40.6 nM) compared to control condition (0 nM). (C) Simulated APD with combined effects of E2 and progesterone at three physiological concentrations corresponding to different stages of the menstrual cycle: early follicular phase (estrogen: 0.1 nM and progesterone: 2.5 nM), late follicular phase (estrogen: 1 nM and progesterone: 2.5 nM) and luteal phase (estrogen: 0.7 nM and progesterone: 40.6 nM). (D) Simulated effects of two physiological concentrations of testosterone (10 and 35 nM) on APD. The corresponding APD at 90% repolarization (APD_90_) is shown in horizontal bar graphs (right panels).

Like humans, many studies have demonstrated that female guinea pigs have slower repolarization than male guinea pigs [1],[36]. To examine the contribution of sex-steroid hormones on the ventricular action potential duration (APD), we included the effects of E2, progesterone and testosterone on membrane currents and simulated action potentials (APs) in three cell types. Figure 2 shows APs for the 50^th^ beat at 1000 ms pacing rate in M cells. Simulated APs of epicardial and endocardial cells are described in Supplemental Figure S2.

E2-induced I_Kr_ suppression contributes to APD prolongation in a dose-dependent manner (Figure 2A). A low concentration of E2 (0.1 nM), corresponding to the early follicular phase of the menstrual cycle, has slight effects on APD compared with control case (from 233 to 235 ms — 0.86% prolongation). However, a concentration of E2 corresponding to the late follicular phase of the menstrual cycle (prior to ovulation) (1.0 nM) prolonged APD (250 ms) by 7.3% (Figure 2A). This value is in good agreement with the observed APD prolongation in guinea pig myocytes in patch-clamp experiments with E2 incubation (11±1%) [25]. Figure 2B shows that progesterone reduced APD in a concentration-dependent manner (222 ms — 4.7% reduction at 2.5 nM corresponding to the follicular phase; 212 ms — 9.0% reduction at 40.6 nM, corresponding to the luteal phase), which agrees with patch-clamp experimental data (6.3% reduction at 40.6 nM) [16].

To investigate the combined effects of E2 and progesterone as they fluctuate during the normal menstrual cycle on the cardiac action potential, we used clinically measured concentrations of hormones at three discrete phases of the menstrual cycle (early follicular, late follicular and luteal). During the early follicular stage, E2 = 0.1 nM, progesterone = 2.5 nM, during the late follicular stage, E2 = 1.0 nM, progesterone = 2.5 nM and during the luteal stage, E2 = 0.7 nM, progesterone = 40.6 nM [23]. As see in Figure 2C, the simulations predict longer APD in the late follicular phase (233 ms) than in the early follicular (223 ms — 4.3% reduction). Simulations predict shortest APD in the luteal phase (218 ms — 6.4% reduction), consistent with experimental observations (≈11% shortening) [36].

We also simulated changes in APD at two physiological concentrations of testosterone (10 nM and 35 nM) shown in Figure 2D, which reflect the normal low and high ranges found in post-pubescent pre-senescent males [21]. The simulations predict marked APD shortening by 10.7% (208 ms) and 15.9% (196 ms) at 10 and 35 nM testosterone, respectively.

### Simulation of tissue-level effects of hormones

**Figure 3:**
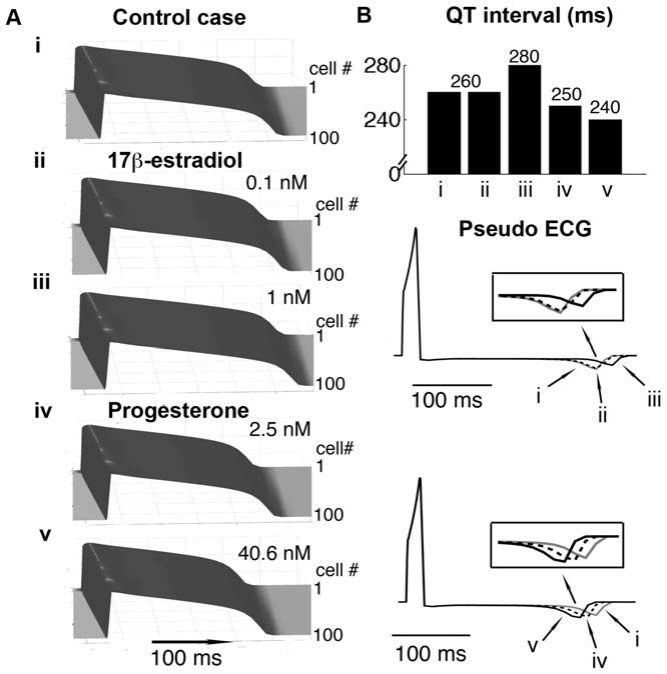
Predicted effects of sex hormones on cardiac tissue and QT-intervals. Action potential (50^th^ paced beat at 1000 ms pacing frequency) propagation from top (cell# 1) to bottom (cell# 100) in a 1 cm cardiac fiber is shown. Time is on the x-axis and voltage on the z-axis. (A) Application of E2 and progesterone (i): control case (no E2), (ii): 0.1 nM E2, (iii): 1 nM E2, (iv): 2.5 nM progesterone, and (v): 40.6 nM progesterone. (B) Comparison of QT intervals is shown in top panel. Lower panels are pseudo ECGs showing the effect of hormones on QT intervals for different cases. The corresponding T-waves are indicated.

We next computed the effects of sex-steroid hormones in a one-dimensional strand of coupled M cells (results from other cell types are shown in Supplemental Figure S2) to determine the effects of hormones in an electrotonically coupled system (Figure 3). We also computed spatial gradients of depolarization and repolarization to generate a pseudo ECG electrogram (Figure 3B). APs were initiated via a stimulus applied to the first cell and then propagated from top to bottom along the 1 cm fiber. Figure 3A show that the first cell fired first and then repolarized first.

The effects of E2 on I_Kr_ leads to dose-dependent APD prolongation in the simulated tissue (Figure 3A), and results in a longer QT interval in the presence of 1 nM (7.7% prolongation) from 260 ms (Figure 3A-i — 0 nM sex-steroid hormone) to 280 ms as seen in Figure 3B (top panel). Also, the simulations clearly show progesterone shortened APD in a dose-dependent manner (3A-iv 2.5 nM, and 3A-v 40.6 nM). The corresponding computed electrograms from the fibers in Figure 3B (lower panel) demonstrates the progesterone-induced QT interval reduction from 260 ms (control case) to 250 ms (3.8% — iv) and 240 ms (7.7% — v).

**Figure 4:**
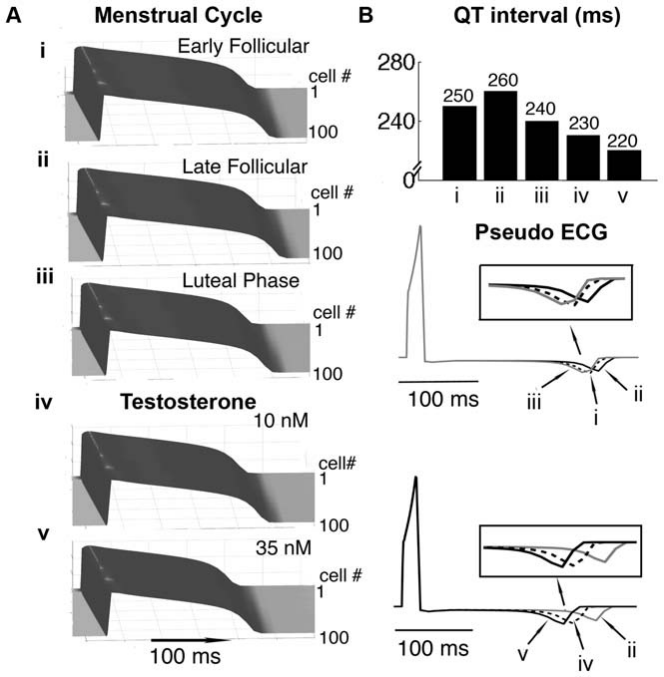
Simulated combined effects of female hormones during the menstrual cycle and male hormones on cardiac action potentials. Shown is the 50^th^ paced beat at a cycle length of 1000 ms in 1D cables. (A) (i): Early follicular phase (ii): Late follicular phase (iii): Luteal phase. Simulated APD in the presence of two physiological concentrations (iv and v) of testosterone. (B) The computed virtual electrograms show QT intervals change during various stages of menstrual cycle and at two concentrations of testosterone (lower panels). The vertical bar graph shows the QT intervals under different circumstances (top panel).

A recent clinical study has observed that the QT intervals fluctuate during the menstrual cycle, suggesting that progesterone may reverse effects of the estrogen-induced QT prolongation [27]. Figure 4A represents the results of simulations in a 1D cable at combined hormone concentrations observed during various phases of the menstrual cycle. Simulations show a QT interval reduction of 10 and 20 ms in the luteal phase compared to the early and late follicular phases, respectively (Figure 4B — top panel), which agree with the clinically observed QT shortening (≈10 ms shortening in the luteal phase compared to the follicular phase) [27]. The models demonstrate that despite the presence of E2 (0.7 nM) during the luteal phase, high progesterone (40.6 nM) results in luteal phase shortening of APD and a QT interval (on the pseudo-ECG) reduction of 4% (from early follicular phase) and 7.7% (from late follicular phase).

The experimental study from Liu et al. suggested the QT intervals were significantly shorter (11.3%) in male than in female rabbits [37]. In Figure 4A-iv and Figure 4A-v, our simulations show the effects of testosterone on APD in simulated one-dimensional tissue. The model predicts that testosterone-induced faster repolarization and caused QT interval reduction to 230 ms (11.5% shortening — case iv) and 220 ms (15.3% shortening — case v) compared with the late follicular phase (260 ms) in Figure 4B. We also ran these simulations in the presence of 10 nM and 35 nM testosterone and 0.1 nM E2, which is estimated as the average circulating concentration of E2 in men [28] (shown in Supplemental Figure S3). In the presence of E2, QT intervals increase by 10 ms, corresponding to 7.7% (10 nM) and 11.5% (35 nM) shortening compared to the late follicular phase in females.

### Effects of estrogen and testosterone on the sensitivity of I_Kr_ to channel block by drugs

**Figure 5:**
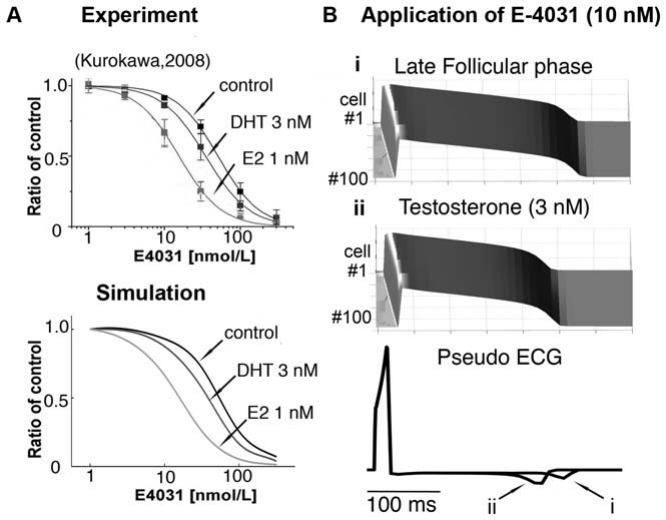
Estrogen and testosterone differentially affect sensitivity of I_Kr_ to drugs. (A) Experimental (top) and simulated (bottom) dose-dependence curves for inhibition of hERG current by E4031 (control — black line), an I_Kr_ blocker, and after addition of estrogen (E2 — light gray line) and DHT (dark gray line). The curves for each concentration of E2 and DHT are indicated. (B) Simulated APD (50^th^ beat at a pacing rate of 1000 ms) with 10 nM E4031 in the presence of both E2 and progesterone (late follicular phase — i). (ii) Simulated effects of testosterone (3 nM) on APD with E4031 application. The computed ECG (low traces) shows that QT interval is substantially longer in case (i) than in case (ii).

Experimental evidence suggests that in the presence of physiological concentrations of E2, the potency of I_Kr_ block by drugs is increased [25]. This finding may explain, in part, the increased susceptibility of females to drug-induced arrhythmias [8],[9]. Hence, we next tested the effect of E2 on I_Kr_ suppression induced by the I_Kr_ channel blocker E-4031 and investigated the effects of female hormones on drug-induced arrhythmia susceptibility. Experimental results [25] shown in Figure 5A (top) illustrate that E2 (1 nM) considerably increased the suppression of hERG by E-4031 (light gray line). However DHT did not greatly change the drug-induced inhibition of hERG current (dark gray line). We then obtained measured ratios of I_Kr_ conductance in the presence of E-4031 and E2 or DHT from the experimental data and used these values to simulate dose-dependence curves for I_Kr_ suppression by E-4031 (control — black line) and after addition of 1 nM E2 (light gray line) and DHT 3 nM (dark gray line) (Figure 5A — lower panel). In Figure 5B (top panel), we show a simulation of a one-dimensional strand of coupled M cells (100 cells) in the late follicular phase during E-4031 treatment, where the model predicts the most dramatic APD and QT interval prolongation. At 10 nM E-4031, the simulated tissue-level APD is shorter with testosterone application (250 ms — 3 nM) compared with APD in the presence of female hormones (280 ms — E2 = 1.0 nM, progesterone = 2.5 nM) as seen in Figure 5B. The pseudo ECG (5B — lower traces) shows that QT interval is substantially longer in the late follicular phase (case i) than with testosterone treatment (case ii).

### Effects of female hormones on pause-dependent LQT-associated arrhythmias with E-4031

**Figure 6:**
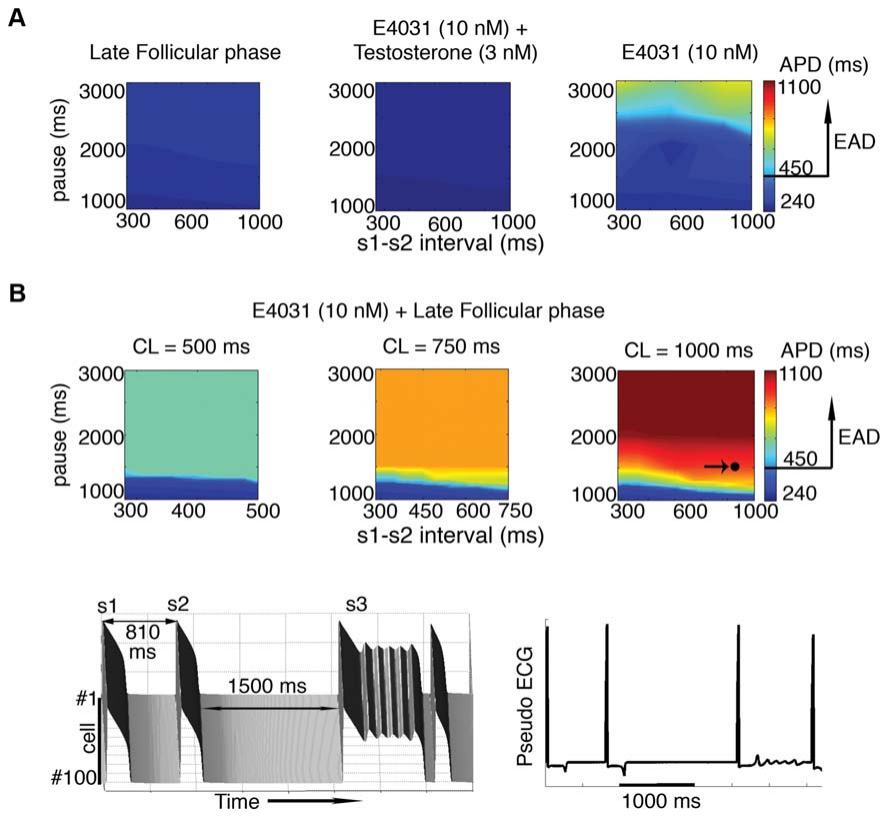
Pause-induced EAD susceptibility is increased in the late follicular phase of the menstrual cycle. (A) The simulated cell was paced for 10 beats at BCL = 1000 ms (s1) followed by varying s1–s2 intervals and long pause intervals. The intervals between s1 and s2 are shown on the x-axis, pause intervals on y-axis and APD are indicated by color gradient. Simulated EAD formations under three conditions, late follicular phase (left panel), in the presence (middle) of testosterone 3 nM and E-4031 10 nM, and addition of E4031 in the absence of sex-steroid hormones (right). (B) Simulated APDs during the late follicular phase with E-4031 (10 nM) application at three basic cycle lengths (500 ms, 750 ms, and 1000 ms). The point indicated by an arrow (right panel) corresponding fiber and pseudo ECG (lower panels) under same conditions.

The exact mechanism of TdP induction is unclear, but it is thought that pause-induced early afterdepolarizations (EADs) can underlie TdP initiation [38],[39]. Hence, we performed a series of simulations to investigate pause-dependent LQT syndrome and its association with arrhythmia susceptibility in the presence of male and female hormones. Single M cells were paced for 10 beats of BCL at 1000 ms (s1) followed by a premature beat (s2) with varying s1–s2 intervals and then a long pause of varying duration as indicated. Our simulations show no EADs (APD>450 ms) occurred during the late follicular phase with no drug application (Figure 6A — left panel) or with the application of E-4031 in the presence of testosterone 3 nM (middle) during a short-long pacing protocol. However in the absence of sex-steroid hormones, EADs were generated by addition of 10 nM E-4031 when the pause interval was very long (>2500 ms) (right panel).

In Figure 6B, we investigated the short-long pacing induced EAD by E-4031 in the late follicular phase, where the concentration of E2 is highest, after pacing at three basic cycle length (500, 750 and 1000 ms). This pacing sequence triggered EADs over a wide range of pauses in all three conditions. APDs of the s3 (post pause) beat are notably lengthened with increasing basic cycle lengths from 500 ms to 1000 ms (Figure 6B — left panel to right panel). Severe EADs were induced at 1000 ms pacing length with a pause greater than 1500 ms (6B — right panel). The point in Figure 6B (right) indicates an EAD that was triggered following a pause of 1500 ms and s1–s2 interval of 810 ms during baseline pacing length of 1000 ms. We have carried out the simulations in a coupled one-dimensional M-cell tissue (6B — lower panels) using the same protocol, and observed propagation of the EAD in the tissue. These simulations suggest that 3 nM testosterone is sufficient to prevent EAD development in the presence of E-4031 10 nM. However, in females, during the late follicular phase of the menstrual cycle, the increased concentration of estrogen appears to exacerbate drug-induced TdP arrhythmias.

### APD prolongation triggers reentrant excitation in heterogeneous tissues

**Figure 7:**
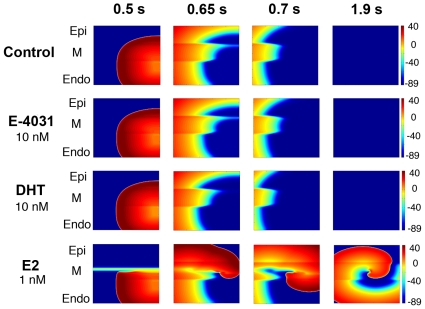
Estrogen increases vulnerability to reentry during short-long-short pacing protocols. Four snapshots following application of hormones and/or drug at indicated time points. Tissues were stimulated along one edge and propagated from endocardial to epicardial region followed by a point stimulus applied in the right corner of the endocardial region. Voltages are indicated by color gradient.

Finally, to test the potential for E2-exacerbated EADs to trigger reentrant arrhythmia activity in 2D heterogeneous tissue, we carried out a series of simulations with varying combinations of hormones and/or drug application. The simulated tissue was stimulated along one edge, and a point stimulus was applied to induce an ectopic beat during a short-long-short sequence described in Supplemental Text S1. Figure 7 shows the results of simulations in four cases at indicated time points. In the absence of hormones or drugs, an initiated wave propagates in all directions, and no reentry occurs (first row). The same behavior is observed following drug application alone (E-4031) and with testosterone application alone (DHT 10 nM). However, when E2 (1 nM) is present (bottom row), the M-cell region is preferentially prolonged (due to the effect of E2 on the background of less repolarizing current that defines this region), which prevents the wavefront from crossing the refractory M-cell region. Instead, the wave propagates leftward at first - until the M-cell region repolarizes, and allows the wave to first cross the M-cell region and then slowly turn to the right. The slowly traveling wavefront (Na^+^ channels are only partially recovered following the prolonged action potential initiated by previous stimulus) begins a cycle of reentry – turning around and continuing to propagate on the wake of the preceding wave (Figure 7 – bottom panel).

**Figure 8:**
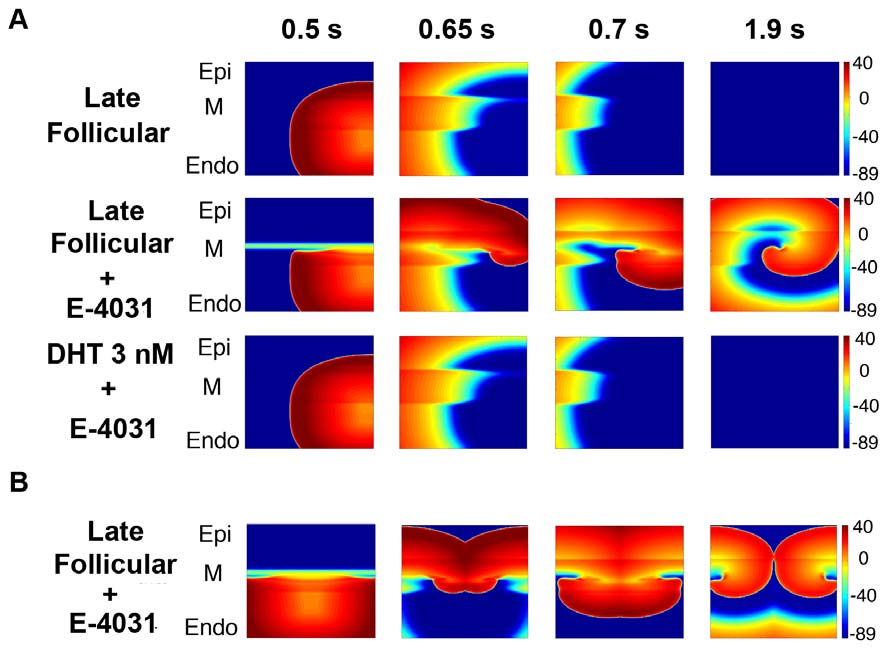
Simulated drug-induced arrhythmias during short-long-short pacing protocols. (A) Comparison of 2D heterogeneous tissue dynamics in the absence or presence of E-4031 during the late follicular phase, and application of testosterone 3 nM with E-4031. (B) The same protocol as above was used, but the premature stimulus was applied during the vulnerable window in the middle of endocardial near the boundary between endocardial region and M cells. The late follicular phase with E-4031 is shown.

In Figure 8A (top), the simulations suggest no reentrant activity during the late follicular phase of the menstrual cycle (progesterone 2.5 nM and 1 nM E2). However, when 10 nM E-4031 is applied during the late follicular phase, a spiral wave is readily induced (Figure 8A – middle). We also tested the effects of male hormone (testosterone) in the presence of E-4031. Figure 8A (bottom) shows that testosterone 3nM with 10 nM E-4031 did not trigger reentry activity.

Interestingly, the induction of a spiral wave in the presence of E-4031 during the late follicular phase of the menstrual cycle is a robust phenomenon. Reentry was introduced in this condition when the ectopic stimulus was applied in the subendocardial or subepicardial region – although not in the M-cell region (not shown). The position of the stimulus is also not critical. Figure 8B shows the effect of a point stimulus applied in the middle of endocardial tissue, leading to the initiation of a pair of counter-rotating spiral waves (Supplemental Figure S4 – protocol 2).

## Discussion

Here, we demonstrate the acute effects of sex steroid hormones in model cells and tissue, from physiological blood concentration to channel interaction, to their effects on APD and tissue dynamics. We used a computational approach to examine the role for acute application of sex steroid hormones on susceptibility to cardiac arrhythmias. The benefit of this approach is that it allows us to investigate the consequences of hormones on cardiac ion channels in isolation, so that observed changes can be specifically attributed to them.

We simulated the acute effects of sex steroid hormones on cardiac cell and tissue dynamics and on fluctuations of QT interval. It has been shown that progesterone enhances I_Ks_, which counterbalances the I_Kr_ reduction by E2. Because estrogen and progesterone dominate in the different phases of menstrual cycle, simulations show that during the late follicular phase (prior to ovulation) of the menstrual cycle, QT interval is longer than in the luteal phase when progesterone is increased, which is consistent with the clinical observation by Nakagawa et al [27]. Notably, the fluctuations in QT interval during the menstrual cycle predicted by our model are within a relatively narrow range of 20 ms, which approximates the clinically assessed standard deviation in pooled QT intervals for a patient population assessed at each phase of the menstrual cycle [31]. One explanation is that such an analysis is unlikely to be sensitive enough to observe significant individual differences in QT intervals as they are fluctuating throughout the menstrual cycle, since biological variability between patients may be larger than fluctuations in individual patients.

Here we demonstrated that increasing testosterone reduced the APD and QT interval in a dose-dependent manner (Figure 2 and 4) by enhancing I_Ks_ and inhibiting I_Ca,L_ current. Moreover, differences in APD become more pronounced between E2 treatment and testosterone treatment when cycle length ≥800 ms - shown in Supplemental Figure S5. Taken all together, these results suggest that sex hormones influence cardiac repolarization in a dose- and cycle length-dependent manner. This is consistent with experimental studies of gender-related differences on cycle length-dependent QT [2],[37].

Since clinical findings suggest female gender is an independent risk factor for TdP arrhythmias and previous experimental studies have shown that E-4031 induced greater prolongation in E2-treated than in DHT-treated animals [2],[37],[40], we investigated the potential for E2 to exacerbate and testosterone to ameliorate arrhythmias in the presence of I_Kr_ block. We used simulations to probe these effects and ask if the presence of these two hormones at physiological concentrations play a key role on gender differences in drug-induced LQTS. We incorporated the experimentally measured combined effects of E2 and E-4031 on I_Kr_ and then simulated them on cardiac tissue dynamics. Since drug-induced TdP is often observed following a short-long-short pacing sequence in clinical studies [38],[41],[42], we explored tissue dynamics using such a protocol. Although we did not observe the development of arrhythmogenic EADs during the late follicular phase of the menstrual cycle (when E2 concentration is at its peak), addition of the I_Kr_ blocker E-4031 resulted in EAD formation in the late follicular phase for a wide range of pacing protocols (Figure 6).

The model simulations also suggested that E-4031 treatment in late follicular phase could lead to initiation of spiral wave reentrant arrhythmias (Figure 8). These simulations imply that at certain phases of the menstrual cycle, elevated levels of E2 may put females at risk for drug-induced arrhythmias – in particular by agents that bind to the promiscuous drug target hERG. Furthermore, we demonstrated that progesterone has a protective effect against E2-induced LQT syndrome (Figure 7 and 8). Spiral waves were not initiated in the presence of low concentrations of progesterone (2.5 nM – late follicular phase). This suggests that progesterone may play an important role in protecting against arrhythmia in females. Unlike the apparent pro-arrhythmic effects of E2 in the presence of I_kr_ block, testosterone was shown in our simulations to prevent E-4031 induced EADs during a short-long-short pacing protocol (Figure 6 and 8).

In the present study, our computational investigation demonstrates the acute effects of progesterone, estradiol and testosterone on cardiac ion channels that are critical for the rate of cardiac repolarization and resultant QT intervals. The models successfully simulates the effects of progesterone and testosterone on cardiac I_Ks_ and I_Ca,L_. These two hormones hasten repolarization, albeit to different extents, and reduce QT interval and susceptibility to LQT-linked arrhythmias. On the contrary, E2 increased QT interval and propensity for TdP arrhythmias by reducing repolarizing current via I_Kr_.

There are several limitations to this study that must be noted. First, we deliberately focused on the effects of acute application of physiological concentrations of sex steroid hormones here, but this means that we have neglected the effects of chronic hormone application. Several studies have suggested that chronic exposure to sex hormones may alter the response of the tissue to acute application of sex hormones [35], alter expression of I_CaL_ (in a species dependent manner) [43]–[47] and may cause structural remodeling of the myocardium [45]. We ran simulations incorporating the measured differences by Verkerk [43] in human I_CaL_ between males and females (since the goal was not to examine guinea pig sex differences). The results of these simulations are in Supplemental Figure S6. As expected, the additional Ca^2+^ current in the female prominently exacerbated the APD prolongation associated with E2. These effects may even be expected to increase in human females where primary repolarizing K^+^ currents, especially I_Ks_, are apparently less prominent than in guinea pig [48]. However, progesterone effects on I_Ks_ in human may offset some of the observed increase in I_CaL_ in human females compared to human males (described above). This issue should be addressed in future studies when the human data are more complete. The guinea pig model is also lacking transient outward K^+^ currents (I_to_) – a subset of channels that have not yet shown to be affected by acute application of sex steroid hormones.

In summary, our study suggests that a computational approach to investigating effects of physiologically circulating hormones can be useful to test and predict their contribution to gender differences in cardiac arrhythmia susceptibility. Moreover, the findings from our model simulations suggest the potential utility of progesterone as a therapeutic agent for inherited and acquired forms of Long-QT Syndrome and that progestin-only contraceptives be given special consideration for their potential amelioration of LQT risk among pre-menopausal women. Finally, the link between estrogen containing hormone replacement therapy among post-menopausal women and increased incidence of adverse cardiac events needs to be investigated in the context of acute hormone effects on ion channels.

## Materials and Methods

The guinea pig cardiac cell model was chosen in this study because we used the guinea pig ventricular myocytes experimental results reported in Ref. 11, 12, and 21. We modified the Faber-Rudy cardiac cell model [49]. The I_Kr_ channel was replaced with Markov model of wild type based on Clancy and Rudy so that E2 effects on activation gating could be readily incorporated. Although the level of detail in terms of description of gating by the I_Kr_ Markov model or the H-H I_Kr_ model is the same, the key difference is that the Markov model takes into account the property of coupling between discrete states, while H-H presumes independence between gating processes. This difference is only relevant in the setting of perturbation to one gating process – precisely what is observed when E2 is present, where activation gating is exhibits a small positive shift in voltage dependence [50]. All the simulation code was in C/C++ and run on Mac Pro 3.0 GHz 8-Core computers. The time step was set to 0.0005 ms during AP upstroke, otherwise the time step was 0.01 ms. Numerical results were visualized using MATLAB R2007a by The Math Works, Inc. Details of computational models and simulation protocols can be found in Supplemental Text S1.

## Supporting Information

Figure S1 Dose-dependence curves are shown for experimental (left traces) and simulated (right traces) enhancement of IKs current by progesterone.
(0.43 MB TIF)

Figure S2 Simulated action potentials for the 50th paced beat at a cycle length of 1000 ms in single epicardial and endocardial cells. The APD for each concentration of sex-steroid hormone is indicated. Epi indicates epicardial; and Endo, endocardial. In epicardial cells, a low concentration of E2 (0.1 nM) has slight effects on APD compared with control case (from 165.73 to 165.22 ms). However, a higher concentration of E2 (1.0 nM) prolonged APD (168.89 ms) (Figure S2A – left panel). For endocardial cells, APD is lengthened by 1 nM E2 from 184.22 ms (0 nM E2) to 189.54 ms. APD is slightly increased to 184.95 ms in the presence of 0.1 nM E2. Figure S2B shows that progesterone reduced APD at 2.5 nM to 157.64 ms in epicardial and 175.79 ms in endocardial cell. At 40.6 nM, progesterone obviously decreases APD to 150.84 ms in epicardial and 168.27 ms in endocardial cell. As see in Figure S2C, the simulations predict longer APD in the late follicular phase (160.79 ms – epi; 180.3 ms – endo) than in the early follicular (158.08 ms – epi; 176.42 ms – endo), and show the shortest APD in the luteal phase (152.82 ms – epi; 171.05 ms – endo). In Figure S2D, we simulated changes in APD at 10 nM and 35 nM of testosterone in epicardial and endocardial cells. The simulations predict marked APD shortening to 149.5 ms (epicardial) and 166.33 ms (endocardial) at 10 nM. At 35 nM testosterone, APDs are reduced to 144.08 ms (epi) and 159.32 ms (endo).
(2.19 MB TIF)

Figure S3 Simulated APD in the presence of two physiological concentrations of testosterone with E2 0.1 nM for the 50th paced beat at a cycle length of 1000 ms in 1D cables. The computed virtual electrograms show QT intervals at two concentrations of testosterone with E2 0.1 nM (lower panel). Our simulations show the effects of testosterone with low concentrations of E2 on APDs in simulated one-dimensional tissue shown in Figure S3-A and B. The models show that testosterone-induced faster repolarization and caused QT interval reduction by 7.7% and 11.5% compared with the late follicular phase in Figure 4A-ii.
(0.57 MB TIF)

Figure S4 Two stimulus protocols were used for 2D heterogeneous cardiac tissue simulations. Red areas indicate stimulus sites.
(2.46 MB TIF)

Figure S5 Action potential durations at 90% repolarization (APD90) are calculated from 100th paced beat at various cycle lengths (between 150 ms and 2000 ms). Sex-steroid hormones alter the action potentials adaptation curves in a concentration-dependent manner. We have demonstrated effects of sex-steroid hormones on APD in cells and tissues, here we calculated action potential duration at 90% repolarization (APD90) from the 100th paced beat at various cycle lengths (between 150 ms and 2000 ms) in order to study gender effects on ventricular reploarization rate. Figure S5 illustrates action potentials adaptation curves for E2, progesterone and the menstrual cycle as well as for two concentrations of testosterone. High concentration of E2 (1 nM) visibly increased APD at cycle lengths longer than 800 ms. In contrast, APD is similar in the control condition (0 nM E2) and a low concentration (0.1 nM) of E2 (Figure S5A). The adaptation curves confirm that predicted effects of E2 on APD90 are larger at longer cycle lengths. On the other hand, 40.6 nM progesterone obviously reduced APD at pacing rates longer than 400 ms (Figure S5B). APD90 was also predicted to have variable cycle length dependence during different phases of the menstrual cycle. At a slow rate APD90 was manifestly longer in the late follicular phase during menstrual cycle than in luteal and early follicular phases as shown in Figure S5C. Figure S5D suggests that testosterone at 10 nM induced marked reduction in APD90 at cycle lengths >400 ms. At 35 nM testosterone, APD90 was additionally shortened.
(2.46 MB TIF)

Figure S6 Figure S6 shows the results of simulations in a 1D cable during the menstrual cycles at combined female hormone concentrations (as described through the paper) and incorporation of a 29% increase in ICa,L in the female case as reported by Verkerk et al., 2005 [43]. Simulations show that during the late follicular phase, EADs were generated on alternate beats while APD shortening occurred in the luteal phase (Figure S6, 50th and 51th beats are shown). Notably, EADs disappeared after 60 beats (not shown), but marked prolongation of APD and QT interval was observed with continued pacing (for 200 beats) in the late follicular phases (B) compared to the early follicular phase (A) and luteal phase (C). The models demonstrate that despite the presence of E2 (0.7 nM) during the luteal phase, high progesterone (40.6 nM) resulted in luteal phase shortening of APD and QT interval.
(1.38 MB TIF)

Text S1 Supplemental figures and methods.
(0.22 MB DOC)
