# Supplementary figures and images for: Acute Effects of Sex Steroid Hormones on Susceptibility to Cardiac Arrhythmias: A Simulation Study
Source: PLoS Comput Biol. 2010 Jan 29;6(1):e1000658. doi: 10.1371/journal.pcbi.1000658 (PMC2813260; doi:10.1371/journal.pcbi.1000658)

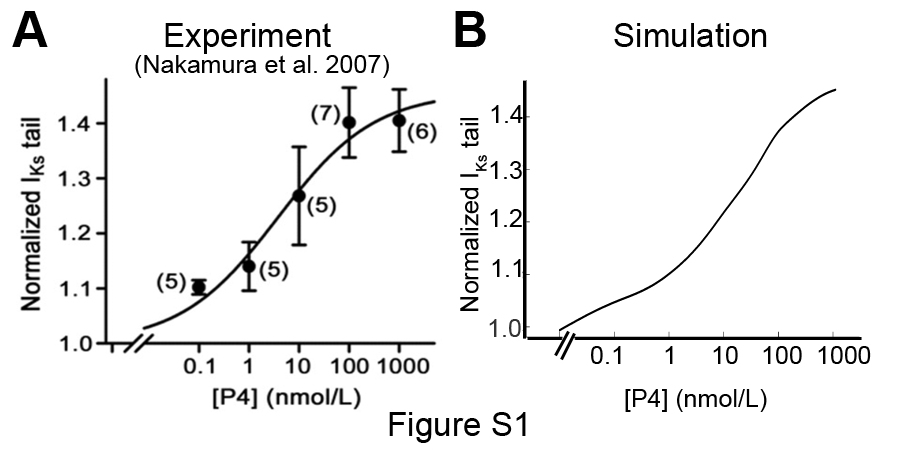

Supplement: Figure S1 — Dose-dependence curves are shown for experimental (left traces) and simulated (right traces) enhancement of IKs current by progesterone. (0.43 MB TIF) [file pcbi.1000658.s001.tif]

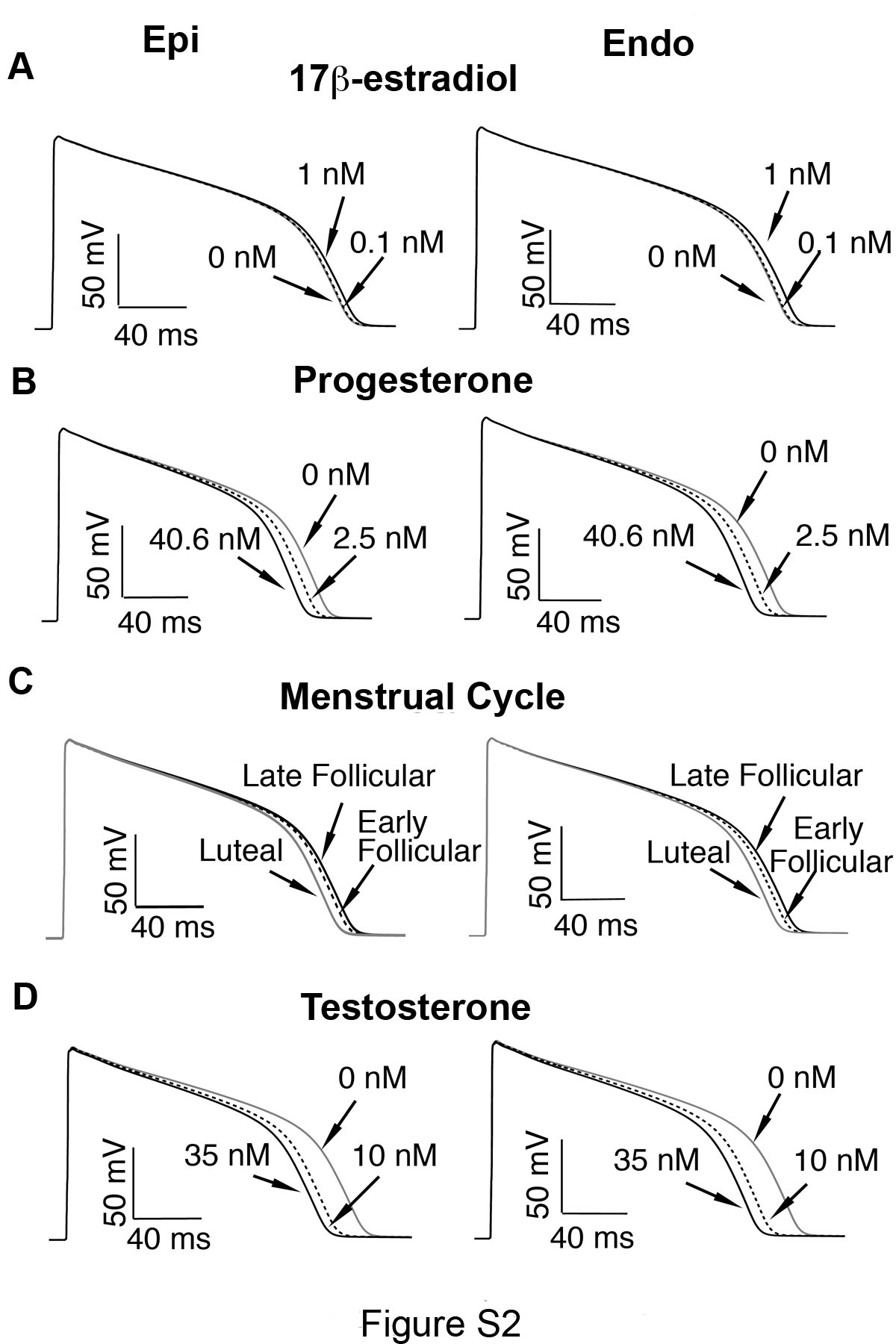

Supplement: Figure S2 — Simulated action potentials for the 50th paced beat at a cycle length of 1000 ms in single epicardial and endocardial cells. The APD for each concentration of sex-steroid hormone is indicated. Epi indicates epicardial; and Endo, endocardial. In epicardial cells, a low concentration of E2 (0.1 nM) has slight effects on APD compared with control case (from 165.73 to 165.22 ms). However, a higher concentration of E2 (1.0 nM) prolonged APD (168.89 ms) (Figure S2A – left panel). For endocardial cells, APD is lengthened by 1 nM E2 from 184.22 ms (0 nM E2) to 189.54 ms. APD is slightly increased to 184.95 ms in the presence of 0.1 nM E2. Figure S2B shows that progesterone reduced APD at 2.5 nM to 157.64 ms in epicardial and 175.79 ms in endocardial cell. At 40.6 nM, progesterone obviously decreases APD to 150.84 ms in epicardial and 168.27 ms in endocardial cell. As see in Figure S2C, the simulations predict longer APD in the late follicular phase (160.79 ms – epi; 180.3 ms – endo) than in the early follicular (158.08 ms – epi; 176.42 ms – endo), and show the shortest APD in the luteal phase (152.82 ms – epi; 171.05 ms – endo). In Figure S2D, we simulated changes in APD at 10 nM and 35 nM of testosterone in epicardial and endocardial cells. The simulations predict marked APD shortening to 149.5 ms (epicardial) and 166.33 ms (endocardial) at 10 nM. At 35 nM testosterone, APDs are reduced to 144.08 ms (epi) and 159.32 ms (endo). (2.19 MB TIF) [file pcbi.1000658.s002.tif]

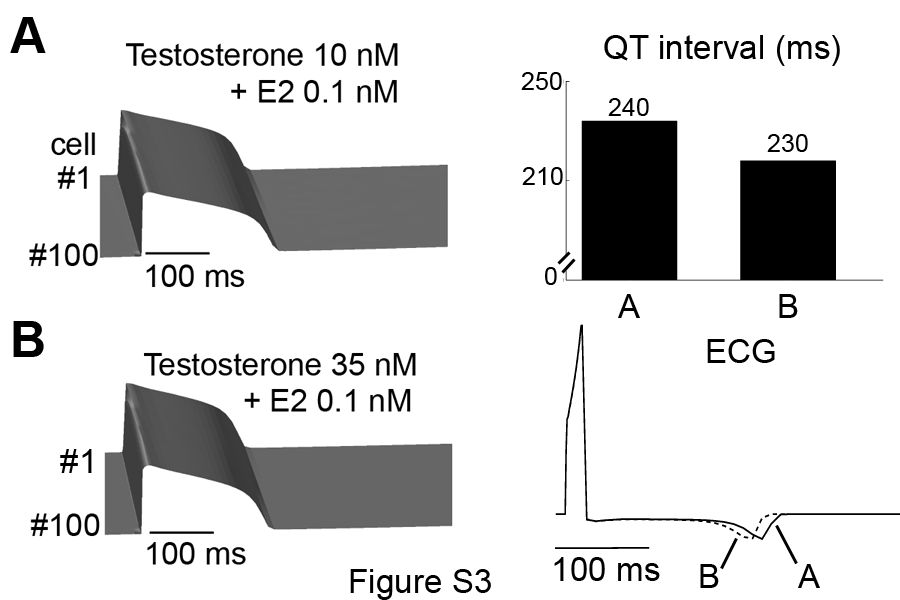

Supplement: Figure S3 — Simulated APD in the presence of two physiological concentrations of testosterone with E2 0.1 nM for the 50th paced beat at a cycle length of 1000 ms in 1D cables. The computed virtual electrograms show QT intervals at two concentrations of testosterone with E2 0.1 nM (lower panel). Our simulations show the effects of testosterone with low concentrations of E2 on APDs in simulated one-dimensional tissue shown in Figure S3-A and B. The models show that testosterone-induced faster repolarization and caused QT interval reduction by 7.7% and 11.5% compared with the late follicular phase in Figure 4A-ii. (0.57 MB TIF) [file pcbi.1000658.s003.tif]

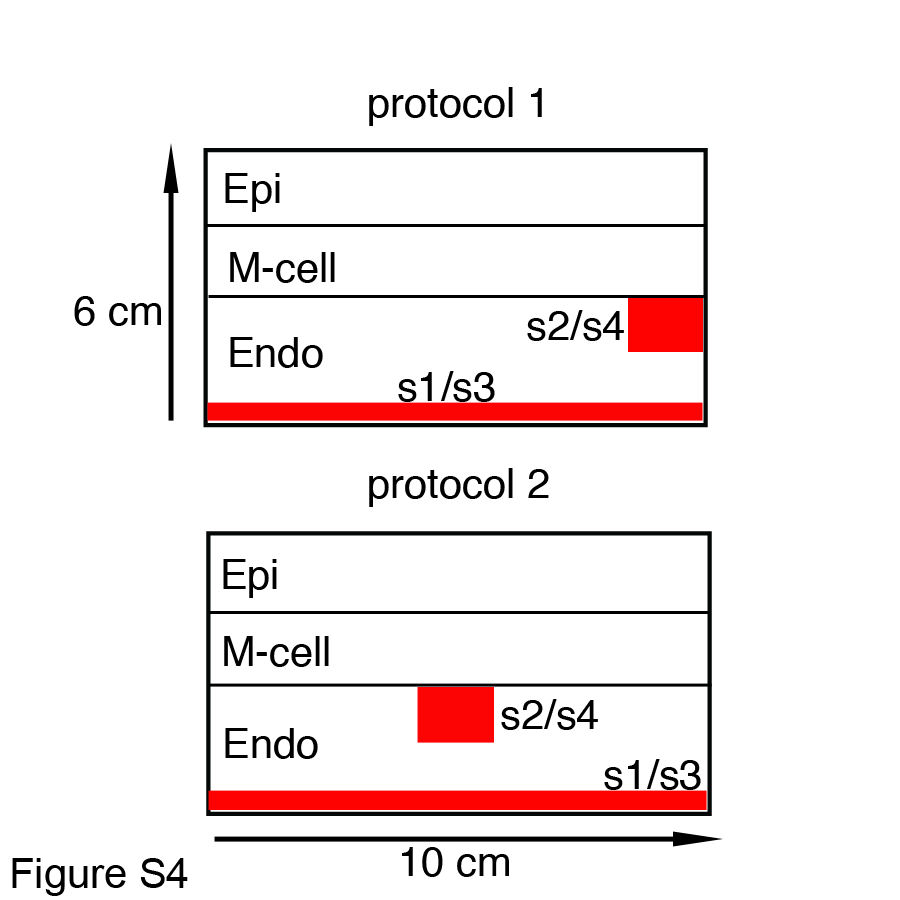

Supplement: Figure S4 — Two stimulus protocols were used for 2D heterogeneous cardiac tissue simulations. Red areas indicate stimulus sites. (2.46 MB TIF) [file pcbi.1000658.s004.tif]

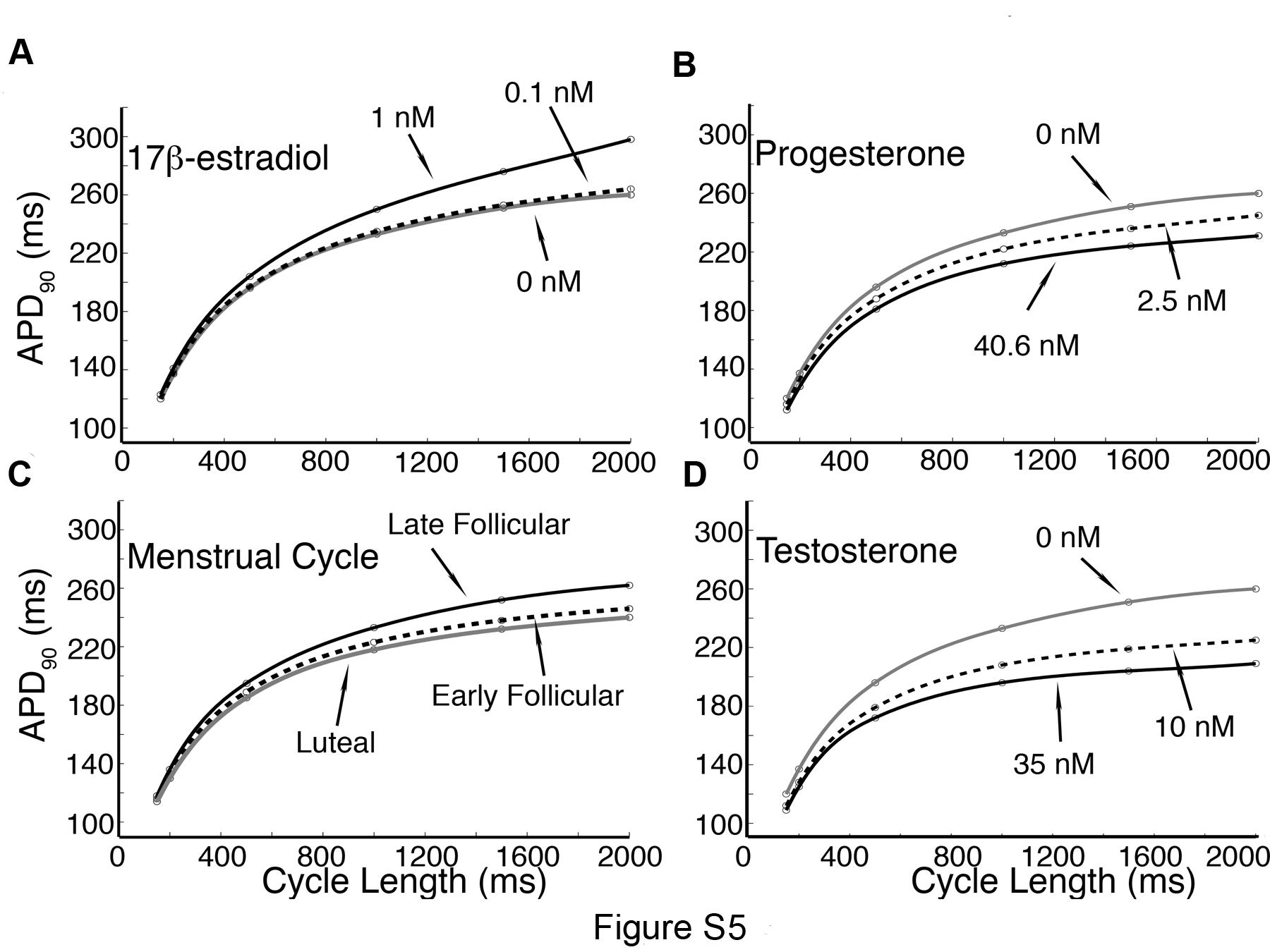

Supplement: Figure S5 — Action potential durations at 90% repolarization (APD90) are calculated from 100th paced beat at various cycle lengths (between 150 ms and 2000 ms). Sex-steroid hormones alter the action potentials adaptation curves in a concentration-dependent manner. We have demonstrated effects of sex-steroid hormones on APD in cells and tissues, here we calculated action potential duration at 90% repolarization (APD90) from the 100th paced beat at various cycle lengths (between 150 ms and 2000 ms) in order to study gender effects on ventricular reploarization rate. Figure S5 illustrates action potentials adaptation curves for E2, progesterone and the menstrual cycle as well as for two concentrations of testosterone. High concentration of E2 (1 nM) visibly increased APD at cycle lengths longer than 800 ms. In contrast, APD is similar in the control condition (0 nM E2) and a low concentration (0.1 nM) of E2 (Figure S5A). The adaptation curves confirm that predicted effects of E2 on APD90 are larger at longer cycle lengths. On the other hand, 40.6 nM progesterone obviously reduced APD at pacing rates longer than 400 ms (Figure S5B). APD90 was also predicted to have variable cycle length dependence during different phases of the menstrual cycle. At a slow rate APD90 was manifestly longer in the late follicular phase during menstrual cycle than in luteal and early follicular phases as shown in Figure S5C. Figure S5D suggests that testosterone at 10 nM induced marked reduction in APD90 at cycle lengths >400 ms. At 35 nM testosterone, APD90 was additionally shortened. (2.46 MB TIF) [file pcbi.1000658.s005.tif]

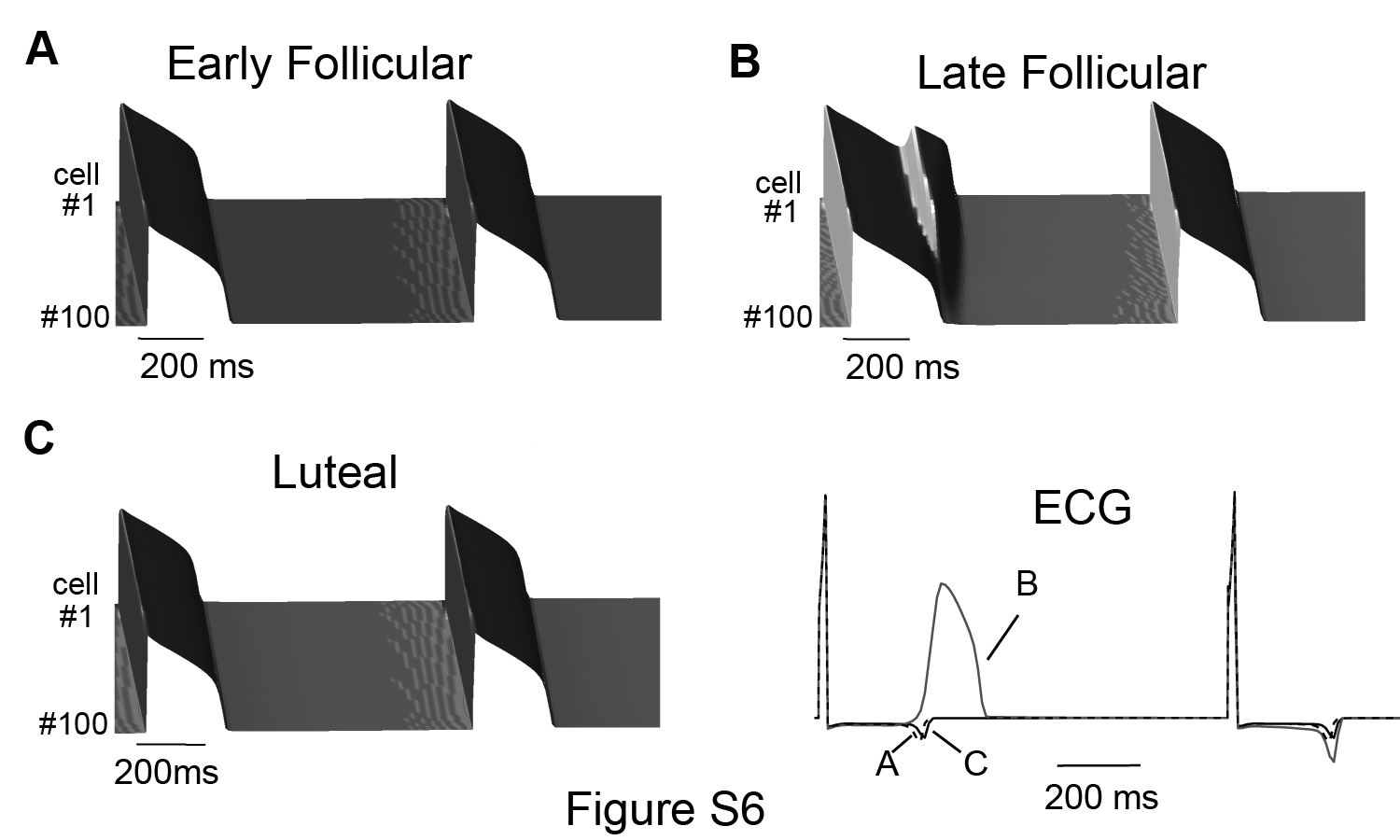

Supplement: Figure S6 — shows the results of simulations in a 1D cable during the menstrual cycles at combined female hormone concentrations (as described through the paper) and incorporation of a 29% increase in ICa,L in the female case as reported by Verkerk et al., 2005 [43]. Simulations show that during the late follicular phase, EADs were generated on alternate beats while APD shortening occurred in the luteal phase (Figure S6, 50th and 51th beats are shown). Notably, EADs disappeared after 60 beats (not shown), but marked prolongation of APD and QT interval was observed with continued pacing (for 200 beats) in the late follicular phases (B) compared to the early follicular phase (A) and luteal phase (C). The models demonstrate that despite the presence of E2 (0.7 nM) during the luteal phase, high progesterone (40.6 nM) resulted in luteal phase shortening of APD and QT interval. (1.38 MB TIF) [file pcbi.1000658.s006.tif]
