# Supplementary material for: Acute Effects of Sex Steroid Hormones on Susceptibility to Cardiac Arrhythmias: A Simulation Study
Source: PLoS Comput Biol. 2010 Jan 29;6(1):e1000658. doi: 10.1371/journal.pcbi.1000658 (PMC2813260; doi:10.1371/journal.pcbi.1000658)
Supplement: Text S1 — Supplemental figures and methods. (0.22 MB DOC) [file pcbi.1000658.s007.doc]

**Text S1. Computational models and simulation protocols**

*Simulation of E2, progesterone and testosterone effects on channels*

We simulated the I_Kr_ current with addition of E2 by scaling the conductance of current and modifying the activation and inactivation curves. In the simulations, we shifted the I_Kr_ activation in the positive direction as experimental results suggested. Currents were scaled to the experimentally measured ratio of conductance: 0.98 for 0.1 nmol/L, 0.9 for 0.7 nmol/L and 0.86 for 1 nmol/L [1].

Rate constants in control case [2]

C1→O or C1→I $\alpha\alpha=65.5\cdot10^{-3}\cdot e^{0.05547153(v-36)}$

O→C1 $\beta\beta=2.9357\cdot10^{-3}\cdot e^{(-0.02158\cdot v)}$

Rate constants in E2 applications

E2 = 0.1 nmol/L:

C1→O or C1→I $\alpha\alpha=65.5\cdot10^{-3}\cdot e^{0.05547153(v-36.3)}$

O→C1 $\beta\beta=2.9944\cdot10^{-3}\cdot e^{(-0.02158\cdot v)}$

E2 = 0.7 nmol/L:

C1→O or C1→I $\alpha\alpha=65.5\cdot10^{-3}\cdot e^{0.05547153(v-38)}$

O→C1 $\beta\beta=3.288\cdot10^{-3}\cdot e^{(-0.02158\cdot v)}$

E2 = 1 nmol/L:

C1→O or C1→I $\alpha\alpha=65.5\cdot10^{-3}\cdot e^{0.05547153(v-39)}$

O→C1 $\beta\beta=3.3761\cdot10^{-3}\cdot e^{(-0.02158\cdot v)}$

Progesterone and testosterone affect the conductance of I_Ks_ but have no distinguishable effects on its kinetics. We used the experimental data [3,4] to determine these values (see full dose-response curve for progesterone both experimentally measured and simulated in Figure S1). When progesterone was applied, we multiplied the conductance of I_Ks_ by scaling factors as follows: for progesterone 2.5 nmol/L: 1.19; for progesterone 40.6 nmol/L: 1.40; for progesterone 100 nmol/L: 1.42. As see in the Figure S1, 50% saturation occurs around 2.5 nM and the effect is nearly saturated at 40.6 nM. In addition, testosterone affects the conductance of I_Ks_ and I_Ca,L_, by experimentally measured ratios as follows: for testosterone 10 nmol/L: I_Ks_ and I_Ca,L_ are 1.38 and 0.94, respectively; for testosterone 35 nmol/L: I_Ks_ and I_Ca,L_ are 1.40 and 0.8, respectively; for testosterone 300 nmol/L: I_Ks_ and I_Ca,L_ are 1.45 and 0.6, respectively.

Under the drug (I_Kr_ blocker  E-4031) condition, the scaling factors for conductance I_Kr_ are: 0.93 with no hormone addition; 0.875 with DHT 3 nM addition; 0.678 with E2 1 nM application [1]. DHT 3 nM affects the conductance of I_Ks_ and I_Ca,L_ by experimentally measured ratios as 1.3 and 0.96 [3].

*One-dimensional simulations*

We simulated a 1 cm fiber composed of 100 ventricular cells connected by resistances to simulate gap junctions [5]. Current flow is described by the following equation:

$$\frac{\partial V(x,t)}{\partial t}=D\frac{\partial^{2}V(x,t)}{\partial x^{2}}-\frac{I_{ion}\_I_{stim}}{C_{m}}$$

Where *V* is the membrane potential, *t* is time, *D* is the tissue diffusion coefficient (0.00092 cm^2^/ms, calculated from Shaw and Rudy [6]), *I_ion_* is the sum of transmembrane ionic currents, *I_stim_* is the stimulus current (300 mA/cm^2^ for 1 ms), and *C_m_* is the membrane capacitance (1 μF/cm^2^). The fiber was paced at BCL = 1000 ms for 50 beats. Action potential simulations were carried out in midmyocardial (M) cells by changing constant of G_Ks_ to 0.125 [7].

A pacing sequence consisted of 10 beats of (s1) basic pacing (500, 750 or 1000 ms) followed by a premature beat (s2) (same cycle-length as s1) and then a delayed beat (s3) delivered after a varying pause (from 1000 to 3000 ms). The stimulus is applied to the first cell.

*ECG computation*

Extracellular unipolar potentials (Φ_e_) generated by the fiber in an extensive medium of conductivity σ_e_, were computed from the transmembrane potential *V_m_* using the integral expression as in Gima and Rudy [8]:

$\Phi_{e}(x©,y©,z©)=\frac{a^{2}\sigma_{i}}{4\sigma_{e}}\int(-\nabla V_{m})\bullet\left[\nabla\frac{1}{r}\right]dx$

$r=[(x-x©)^{2}+(y-y©)^{2}+(z-z©)^{2}]^{1/2}$

where ∇*V* is the spatial gradient of *V_m_*, *a* is the radius of the fiber, *σ_i_* is the intracellular conductivity, *σ_e_* is the extracellular conductivity, and *r* is the distance from a source point (x, y, z) to a field point (x’, y’, z’). *Φ_e_* was computed at an “electrode” site 2.0 cm away from the distal end along the fiber axis.

*Two-dimensional simulations*

$$\frac{\partial V(x,y,t)}{\partial t}=D_{x}\frac{\partial^{2}V(x,y,t)}{\partial x^{2}}+D_{y}\frac{\partial^{2}V(x,y,t)}{\partial y^{2}}-\frac{I_{ion}\_I_{stim}}{C_{m}}$$

Where *V* is the membrane potential, *x* and *y* are distances in the longitudinal and transverse directions, respectively, D_x_ and D_y_ are diffusion coefficients in the x and y directions, and all other parameters are as in the one-dimensional simulations. *I_stim_* is 80 mA/cm^2^ for 1 ms. We simulated a heterogeneous cardiac tissue on 600 by 1000 with Δx = Δy = 100 μm. The tissue contains an endocardial region (fibers 1 to 240), M-cell region (fibers 241 to 420) and epicardial region (fibers 421 to 600). In guinea pig heart, the density ratio of I_Ks_ to I_Kr_ is about 23:7:15 in epi-/mid-/endocardial cells [7]. The exact scaling constants of G_Ks_ for epicardial, mid- and endocardial cells are 0.433, 0.125 and 0.289, respectively. We also incorporated anisotropic effects by setting D_x_ and D_y_ such that the longitudinal and transverse conduction velocities are 0.56 m/s and 0.26 m/s, respectively.

The 2D tissue was simulated using a typical short-long-short protocol [9,10]. The tissue was first paced for 10 beats (s1) at BCL = 1000 ms on the entire length of one side of tissue. A premature stimulus (s2) was then applied in a 1 cm × 1 cm area on the right edge of the endocardial region and then a delayed beat (s3) was delivered after a long pause (1700 ms). Following the s3, another premature beat (s4) was applied in the same square region as s2 to simulate a short-long-short ventricular cycle (Figure S4 – protocol 1).

**References**

1. Kurokawa J, Tamagawa M, Harada N, Honda SI, Bai CX, et al. (2008) Acute effects of estrogen on the guinea pig and human IKr channels and drug-induced prolongation of cardiac repolarization. J Physiol.

2. Clancy CE, Rudy Y (2001) Cellular consequences of HERG mutations in the long QT syndrome: precursors to sudden cardiac death. Cardiovasc Res 50: 301-313.

3. Bai CX, Kurokawa J, Tamagawa M, Nakaya H, Furukawa T (2005) Nontranscriptional regulation of cardiac repolarization currents by testosterone. Circulation 112: 1701-1710.

4. Nakamura H, Kurokawa J, Bai CX, Asada K, Xu J, et al. (2007) Progesterone regulates cardiac repolarization through a nongenomic pathway: an in vitro patch-clamp and computational modeling study. Circulation 116: 2913-2922.

5. Faber GM, Rudy Y (2000) Action potential and contractility changes in [Na(+)](i) overloaded cardiac myocytes: a simulation study. Biophys J 78: 2392-2404.

6. Shaw RM, Rudy Y (1997) Electrophysiologic effects of acute myocardial ischemia. A mechanistic investigation of action potential conduction and conduction failure. Circ Res 80: 124-138.

7. Viswanathan PC, Shaw RM, Rudy Y (1999) Effects of IKr and IKs heterogeneity on action potential duration and its rate dependence: a simulation study. Circulation 99: 2466-2474.

8. Gima K, Rudy Y (2002) Ionic current basis of electrocardiographic waveforms: a model study. Circ Res 90: 889-896.

9. Liu J, Laurita KR (2005) The mechanism of pause-induced torsade de pointes in long QT syndrome. J Cardiovasc Electrophysiol 16: 981-987.

10. Yan GX, Wu Y, Liu T, Wang J, Marinchak RA, et al. (2001) Phase 2 early afterdepolarization as a trigger of polymorphic ventricular tachycardia in acquired long-QT syndrome : direct evidence from intracellular recordings in the intact left ventricular wall. Circulation 103: 2851-2856.
